# Reply to: Prognostic significance of gastrointestinal dysfunction in critically ill patients with COVID-19

**DOI:** 10.62675/2965-2774.20250233

**Published:** 2025-10-27

**Authors:** Ricardo Antônio Correia Lima, Annika Reintam Blaser, Júlia Falconiere Paredes Ramalho, Barbara Cristina de Almeida Campos Lacerda, Gabriela Sadigurschi, Paula Fonseca Aarestrup, Rafael Aguilar Sales, João Mansur, Roberto Muniz Ferreira

**Affiliations:** 1 Hospital Samaritano Intensive Care Unit Rio de Janeiro RJ Brazil Intensive Care Unit, Hospital Samaritano - Rio de Janeiro (RJ), Brazil.; 2 Universidade Federal do Estado do Rio de Janeiro Department of General Surgery Rio de Janeiro RJ Brazil Department of General Surgery, Universidade Federal do Estado do Rio de Janeiro - Rio de Janeiro (RJ), Brazil.; 3 University of Tartu Department of Anaesthesiology and Intensive Care Tartu Estonia Department of Anaesthesiology and Intensive Care, University of Tartu - Tartu, Estonia.; 4 Lucerne Cantonal Hospital Department of Intensive Care Medicine Lucerne Switzerland Department of Intensive Care Medicine, Lucerne Cantonal Hospital - Lucerne, Switzerland.; 5 Universidade Federal do Rio de Janeiro Edson Saad Heart Institute Rio de Janeiro RJ Brazil Edson Saad Heart Institute, Universidade Federal do Rio de Janeiro - Rio de Janeiro (RJ), Brazil.; 6 Hospital Samaritano Department of Cardiology Rio de Janeiro RJ Brazil Department of Cardiology, Hospital Samaritano - Rio de Janeiro (RJ), Brazil.

**To the Editor**

We appreciate the constructive comments provided by Finsterer et al.^(1)^ in the letter to the editor concerning our manuscript titled "Prognostic significance of gastrointestinal dysfunction in critically ill patients with COVID-19".^(2)^ We have attempted to elucidate all the concerns by addressing each argument individually.

The inherent limitations of retrospective studies are well defined, and any research that utilizes such a design must be interpreted within the boundaries of what the data can provide. However, there are specific scenarios where retrospective analyses are essential and sometimes the only option to guide further research in the field. Our study was developed during the most demanding phase of the coronavirus disease 2019 (COVID-19) pandemic, when little was known about the disease and worldwide resources were limited. Several retrospective studies were performed during that time, which were vital to describe the prognostic determinants associated with the natural course of the disease, many of which are still currently utilized for decision-making.^(3–5)^ The study was not designed to have continuity on its own, but rather to provide hypothesis-generating information that could be utilized to develop future investigations.

All consecutive patients admitted to the intensive care unit (ICU) due to COVID-19 during the study period were screened for inclusion. During the pandemic, the information recorded in the medical charts of ICU patients at our institution was standardized and thorough to provide as much knowledge as possible concerning the disease, as reflected in multiple subsequent publications derived from our database.^(6–8)^ There were no exclusions due to missing data, and no variable provided in the manuscript was missing from any of the included patients.

Gastrointestinal (GI) compromise extends beyond the manifestation of isolated and self-limited symptoms and has been associated with adverse clinical outcomes among COVID-19 patients and many other diseases, as reported by previous publications and our manuscript.^(9)^ We did not hypothesize or assert that COVID-19 increased the risk of death specifically due to GI complications, as no patient in the study actually developed life-threatening GI manifestations, such as mesenteric ischemia or intestinal obstruction. Alternatively, given the systemic nature of COVID-19, GI dysfunction most likely serves as a marker of disease severity, which may have explained the possible relationship with respiratory compromise and death. These associations remained even after controlling for variables representing the pathophysiological triad of severe COVID-19: hemodynamic compromise (systolic blood pressure), respiratory failure (oxygen saturation), and systemic inflammation (C-reactive protein). Previous studies have also reported similar results.^(9)^

We recognize that GI dysfunction is associated with many different conditions, and not all manifestations recorded in the study may have directly resulted from the infection. However, regardless of the underlying cause, our data suggests that in the context of severe COVID-19, a flare-up of GI dysfunction could have prognostic implications, as observed in other critically ill patients.^(10)^ Analysing how specific causes of GI disease interact with severe COVID-19 would require a significantly greater number of patients and a distinct study design. Additionally, considering the limited diagnostic resources and high risk of in-hospital viral spreading during the study period, further etiological investigations of possible GI comorbidities were not warranted, especially since most symptoms were mild.

The correct classification of causes of death is still a challenge in medical research, most notably when specific criteria are not defined, as in most observational studies.^(11,12)^ During the initial stages of the pandemic, deaths may have occurred without a thorough clinical investigation of underlying conditions, possibly introducing bias regarding specific causes. Since GI dysfunction was not hypothesized in our study as a significant direct cause of death, its clinical significance would have been underestimated if such an analysis were performed in isolation. For this reason, total death was chosen as the study's primary outcome, minimizing the risk of assessment bias by not limiting the analysis specifically to GI-related deaths when associating GI dysfunction with a poor prognosis. Any prognostic condition that is associated with total death has significant clinical value and should not be overlooked.

The GI dysfunction score was calculated using data collected from the second to the seventh day of ICU stay. Patients who died or were discharged before the seventh day were still included in the analysis, considering information derived from the number of days they remained in the ICU after the first 24 hours. This was established to exclude patients who may have died in the first 24 hours, and to minimize the influence of confounding complications associated with longer lengths of ICU stay. Furthermore, this period was optimized to consider the prognostic influence of GI dysfunction during COVID-19's cytokine storm, representing the most critical phase of the disease's natural course. Though patients may have developed GI dysfunction after leaving the ICU, it would have occurred subsequently to the aforementioned stage, and thus not in a phase of critical illness, as specified in the manuscript's title and objectives. The development of GI dysfunction after hospital discharge is also a known complication within the context of long COVID syndrome, and was not the scope of our study.^(13)^

The year 2020 will be remembered as a period of significant paradigm shifts across many scientific fields and medical specialties. The COVID-19 pandemic imposed an unprecedented burden on several governments, health systems, and the entire scientific community around the World. As researchers relentlessly worked to learn about the disease, observational studies were the mainstay of scientific evidence, and many were performed with a retrospective design. Unveiling the many different pathophysiological features of COVID-19 was essential for orienting future research and ultimately implementing effective preventive and therapeutic measures. We agree with Finsterer et al.^(1)^ that all medical research should undergo critical appraisal according to the foundations of evidence-based medicine, which is invaluable for scientific progress and improvement in patient outcomes. Our results should also be perceived with such scrutiny. However, despite the inevitable limitations of retrospective studies, hopefully our work has revealed new possibilities for exploring the important topic of GI dysfunction in the context of severe COVID-19 and perhaps of other acute respiratory viral illnesses that may arise in the future.
